# Cause rare d'une perforation de la cloison nasale

**DOI:** 10.11604/pamj.2014.17.23.3358

**Published:** 2014-01-17

**Authors:** Ali Jahidi, Bouchaïb Hemmaoui, Wulfran Rosaire Itoua, Noureddine Errami, Fouad Benariba

**Affiliations:** 1 Service ORL hôpital militaire d'instruction Mohammed V, Rabat, Maroc

**Keywords:** Tuberculose, cloison nasale, perforation, biopsie, Tuberculosis, nasal septum, perforation, biopsy

## Abstract

Les perforations de la cloison nasale constituent une pathologie fréquente en ORL. Leurs causes sont multiples et restent dominées par les traumatismes chirurgicaux. Les étiologies infectieuses et notamment la tuberculose sont rares. Nous présentons le cas d'une patiente ayant bénéficié d'une méatotomie bilatérale et chez laquelle une perforation de la cloison nasale a été découverte lors d'un examen systématique à un an de son intervention. D'abord considérée comme une complication de la chirurgie, la biopsie des berges de la perforation a permis de déterminer son origine tuberculeuse. La présentation clinique des perforations de la cloison nasale n'est pas spécifique. Elles sont souvent asymptomatiques et de découverte fortuite. Les traumatismes notamment chirurgicaux sont le plus siuvent en cause. L′orogine tuberculeuse resta très rare. Le diagnostic de certitude de tuberculose repose sur la biopsie des berges de cette perforation. Le but de ce travail est de mettre en avant l'intérêt de la biopsie dans le diagnostic de la tuberculose de la cloison nasale. Cette biopsie doit être systématique même en cas d'antécédents de chirurgie endonasale qui est le plus souvent en cause dans les perforations de la cloison nasale.

## Introduction

La tuberculose est un problème grave de santé dans le monde qui touche de nombreuses personnes dans les pays en développement. L′épidémie de syndrome d′immunodéficience acquise et le développement de souches résistantes aux antibiotiques anti- mycobactéries ont contribué à l′incidence croissante internationale de la tuberculose ces dernières années. L'infection touche typiquement les poumons, et les localisations extra pulmonaires restent relativement peu fréquentes [1]. Dans ce cadre La tuberculose nasale est une infection chronique rare qui peut être primaire par inhalation ou bien secondaire à une autre localisation à distance [1, 2] A travers une observation clinique et une revue de la littérature, nous discutons les difficultés diagnostic et thérapeutique d'un cas de tuberculose nasale primaire révélée par une perforation de la cloison nasale.

## Patient et observation

Une femme de 64 ans a consulté il y a environ un an pour une symptomatologie rhinologique faite d'obstruction nasale bilatérale, d'abord intermittente puis devenue progressivement quasi permanente, d'une rhinorrhée antérieure muco-purulente, d’éternuements et d'une épistaxis de faible abondance particulièrement après un mouchage intempestif. Dans les antécédents de la patiente on retrouvait une chirurgie endonasale type méatotomoie moyenne bilatérale (selon le compte rendu opératoire) pour une sinusite maxillaire chronique realisée une année avant la premiere consultation.

**Figure 1:**
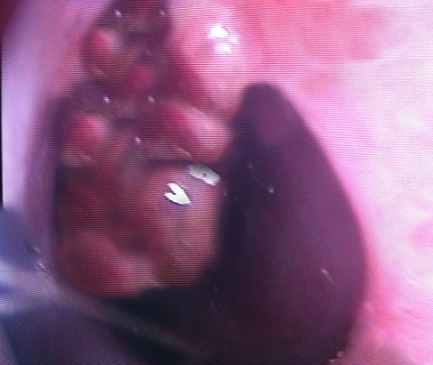
Perforation de la cloison nasale avec secrétions et croutes sales avant traitement

L'examen clinique ORL et notamment des fosses nasales trouvait une hypertrophie des cornets inférieurs qui étaient recouverts de secrétions purulente et une perforation antéro-inférieure de la cloison nasale à bords irréguliers contenant des croutes brunâtres et des stigmates de saignement récent (Figure 1)

Cette perforation, apparue chez cette patiente aux antécédents de chirurgie endonasale a d'emblée été liée à un traumatisme chirurgical, en l'absence d'autre cause évidente. Mais la persistance du caractère sale et crouteux des berges de la perforation malgré des traitements antibiotiques et antiseptiques bien menés associés à des lavages quotidiens, nous a poussé à soulever l'hypothèse d'une infection à germes spécifique ou d'une granulomatose et à réaliser des biopsies aux bords de la perforation. L'examen anatomopathologique a montré une muqueuse de type respiratoire reposant sur un chorion où siège de nombreux granulomes épithéloides et giganto-cellulaire avec une nécrose caséeuse.

**Figure 2:**
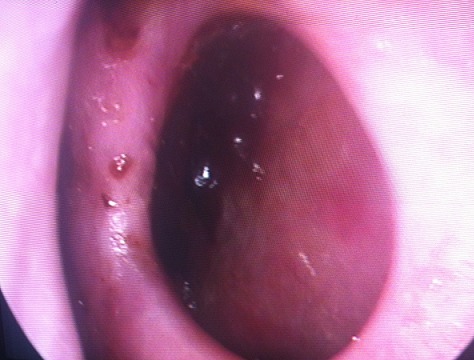
Perforation de la cloison nasale propre après traitement

Le diagnostic de perforation de la cloison nasale d'origine tuberculeuse a été retenu, et la recherche d'autres localisations de tuberculose a été entamée et s'est révélée négative confirmant ainsi le caractère primaire de cette localisation tuberculeuse. La patiente a été adressée au service de pneumophtysiologie où elle a reçu un traitement anti-bacillaire à base d'isoniazide, rifampicine et pyrazinamide pendant 2 mois puis d'isoniazide et de rifampicine pendant 7 mois. L’évolution a été marquée par une nette amélioration clinique et la disparition des épistaxis et de l'aspect crouteux des bords de la perforation, dont le diamètre est resté sensiblement le même (Figure 2).

## Discussion

La tuberculose semble, a ce jour, encore progresser dans le monde et selon un rapport de l'organisation mondiale de la santé elle reste la principale cause de décès parmi toute les maladies infectieuse chez l'Homme [1]. Elle est causée par des bactéries appartenant au complexe Mycobacterium tuberculosis et affecte généralement les poumons, Bien que dans près d′un tiers des cas, d′autres organes peuvent également être atteints. Ainsi la localisation au niveau de la muqueuse nasale est rare [1]. Elle peut être primaire par inhalation ou inoculation directe par un doigt contaminé [2, 3] ou bien secondaire à une autre localisation à distance [4]. Sa rareté pourrait être expliquée par la protection secondaire au mouvement ciliaire de la muqueuse nasale associée à l'action bactéricide du mucus nasal [2] ainsi que le rôle de filtre joué par les vibrisses nasales.

Dans une revue de 35 cas de tuberculose nasale Butt et al [5] retrouvent une prédominance féminine et principalement chez des personnes âgées, les sites les plus fréquemment touchés étant le septum nasal suivi par le cornet inférieur. La symptomatologie rencontrée n'est ni très riche, ni évocatrice et peut prendre le tableau clinique d'une rhinite chronique [2]. nous retrouvons par ordre décroissant de fréquence Selon la littérature [6], épistaxis (54-74%), présence de croûtes (48-91%), obstruction nasale chronique(47-56%), sifflement (18-19%), rhinorrhée (6-8%), douleurs (7%), céphalées (7%), hyposmie (3-4%) ou modification de la voix (2%) il est communément admis qu'il existe une corrélation entre une taille croissante, le positionnement antérieur de la perforation et sa symptomatologie [7]. Mais Aucun de ces symptômes n'est spécifique de l'atteinte tuberculeuse. Le cas que nous présentons conforte cette tendance car il s'agit d'une femme de 64 ans qui a consulté pour une symptomatololgie atypique et trainante. Il faut noter cependant que la perforation de la cloison nasale indépendamment de sa cause est souvent asymptomatique et de découverte fortuite lors d'une consultation pour un autre motif [6, 8].

Le diagnostic de certitude de l'origine tuberculeuse d'une perforation de la cloison nasale repose sur la biopsie des berges de la perforation qui met en évidence la présence de granulome et de nécrose caséeuse. Il faut toutefois se rappeler qu'il n'est pas rare que ces biopsies reviennent, à tort, négatives. Cette négativité pouvant s'expliquer par l'exigüité des prélèvements et leur réalisation dans des zones de nécrose [9]. Il est donc important de pratiquer des biopsies à la jonction du tissu sain et du tissu pathologique et de les répéter sur toutes les zones suspectes [10]

Le diagnostic différentiel se pose avec les autres lésions granulomateuses comme la granulomatose de wegner, la sarcoïdose, la syphilis ou la leishmaniose [2]. Le traitement est essentiellement médical à base d'anti-bacillaires: Isoniazide, Rifampicine, Pyrazinamide et streptomycine ou Ethambutol pendant 6 à 9 mois en fonction de l’évolution [2, 11]

## Conclusion

Les perforations de la cloison nasale constituent une pathologie fréquente en ORL. La plupart sont, ou demeurent, longtemps asymptomatiques. Si elles relèvent essentiellement des causes traumatiques locales et notamment la chirurgie endonasale, Elles peuvent aussi accompagner, voire révéler, de nombreuses maladies systémiques et plus rarement une maladie infectieuse chronique comme la tuberculose. La perforation signant dans ce cas la gravité et l'ancienneté de la maladie [1]. Il faut donc garder à l'esprit, notamment dans les pays en voie de développement ainsi que dans certains pays occidentaux qui connaissent un afflux important de migrants venant de régions endémiques, que la réalisation de biopsies des berges d'une perforation de la cloison nasale doit être systématique pour poser le diagnostique de cette maladie aujourd'hui parfaitement curable; même en cas d'antécédent de chirurgie endonasale dont la réputation de première pourvoyeuse de perforation septale peut constituer un véritable piège pour le praticien.
